# The Characterization of the Purine Nucleoside Phosphorylase from *Agaricus bisporus* and Its Potential Application in Reducing Purine Content in Beer

**DOI:** 10.3390/jof11040268

**Published:** 2025-03-31

**Authors:** Jun Liu, Jian Lu

**Affiliations:** 1 Key Laboratory of Industrial Biotechnology, Ministry of Education, School of Biotechnology, Jiangnan University, Wuxi 214122, China; liujunchina@gdaas.cn; 2 National Engineering Research Center of Cereal Fermentation and Food Biomanufacturing, Jiangnan University, Wuxi 214122, China; 3 Jiangsu Provincial Research Center for Bioactive Product Processing Technology, Jiangnan University, Wuxi 214122, China; 4 Sericultural and Agri-Food Research Institute, Guangdong Academy of Agricultural Sciences, Key Laboratory of Functional Foods, Ministry of Agriculture and Rural Affairs, Guangdong Key Laboratory of Agricultural Products Processing, Guangzhou 510610, China; 5 Key Laboratory for Prevention and Control of Avian Influenza and Other Major Poultry Diseases, Ministry of Agriculture and Rural Affairs, Key Laboratory of Livestock Disease Prevention of Guangdong Province, Institute of Animal Health, Guangdong Academy of Agricultural Sciences, Guangzhou 510640, China

**Keywords:** beer, gout, purines, *Agaricus bisporus*, purine nucleoside phosphorylase

## Abstract

Beer, the most popular alcoholic beverage, poses health risks for individuals with gout and hyperuricemia due to its high purine content. Herein, we identified a novel purine nucleoside phosphorylase (*Ab*PNP) from the edible mushroom *Agaricus bisporus* and heterologously expressed it in *Pichia pastoris*. The recombinant *Ab*PNP exhibited optimal activity at 60 °C and pH 7.0, retaining >80% activity at pH 6.0–9.0 and >85% activity after 3 h at ≤60 °C. Kinetic analysis revealed high catalytic efficiency (kcat/Km = 2.02 × 10^6^ s^−1^⋅M^−1^) toward inosine, with strong resistance to metal ions except for Co^2+^ and Cu^2+^. The application of *Ab*PNP (1.0–5.0 U/mL) during wort saccharification reduced purine nucleosides by 33.54% (from 151.53 to 100.65 mg/L) while increasing yeast utilization of free purine bases. The resulting beer showed improved fermentation performance (alcohol content increased by 3.6%) without compromising flavor profiles. This study provides the food-grade enzymatic strategy for low-purine beer production, leveraging the GRAS status of both *A. bisporus* and *P. pastoris*.

## 1. Introduction

Gout is among the most prevalent inflammatory joint diseases, characterized by hyperuricemia. The literature has indicated that it is caused by purine metabolism disturbances or impaired uric acid excretion by the kidneys [1]. Alcoholic beverages contain purine-like substances and alcohol, which are the main triggers of gout. When the blood alcohol levels are above 200 mg/dL, the lactic acid content in the blood increases due to the oxidation process of ethanol, which inhibits renal uric acid excretion, disrupts the original balance, and increases the risk of gout [2]. Health professionals have revealed that beer is an independent factor associated with the development of gout, with a higher risk for beer than for spirits [3]. However, moderate alcohol consumption does not increase the risk of gout. Furthermore, low-malt and low-purine beers have a smaller effect on blood uric acid concentrations than many other beers [4]. Although non-alcoholic beers contain very little or no alcohol, they can also elevate serum uric acid levels. Therefore, in beers, the purine content, not the alcohol content, is associated with a high risk of gout. During the beer brewing, the wort produced by the saccharification process contains many purine nucleosides and free purine bases. During fermentation, the yeast also uses free purine bases for proliferation, which produces bound purine nucleosides, with a small number of free purine bases remaining in the beer. In China, the total purine content of beer ranges from nearly 30 to 150 mg/L, averaging 74.89 mg/L [5].

Gout can be treated by either drug therapy or diet therapy [6]. The former can alleviate symptoms, reduce uric acid production, promote uric acid excretion, and induce uric acid catabolism in four ways, such as colchicine, non-steroidal anti-inflammatory drugs, glucocorticoids, and adrenocorticotropic hormones. However, these anti-gout agents often have substantial side effects, such as hepatitis, gastrointestinal bleeding, and other symptoms, including hypersensitivity reactions. Dietary therapy includes reducing exogenous purine intake through a dedicated low-purine diet, which decreases uric acid production in the body to some extent while effectively avoiding toxic side effects.

Since it is challenging to restrict diet, enzymatically decreasing purine levels in popular beer provides a safe and efficient strategy to control hyperuricemia [7]. Before selecting an appropriate purine-degrading enzyme, it is essential to understand the forms of purine analogs present in beer. By tracing the changes in purine nucleoside and free purine content during the fermentation of 12.5 ºP wort [8], the authors observed that free purine levels rapidly decreased from 70 to 10 mg/L in the early stages of fermentation, while the total purine nucleoside content remained stable at 90 mg/L. This suggests that the purines in beer are primarily present in the form of purine nucleosides rather than free purine bases.

Purine nucleoside phosphorylase (PNP) (EC 2.4.2.1) is the primary enzyme in the purine salvage pathway [9]. In the presence of orthophosphate as a second substrate, PNP catalyzes the reversible phosphorolytic cleavage of the glycosidic bond of purine nucleosides (and some analogs): purine nucleoside + orthophosphate ↔ purine base + α-d-pentose-1-phosphate. Furthermore, PNP degrades the purine nucleosides in the wort into free purine bases, which are then utilized by the yeast during fermentation to minimize the beer’s purine content. In a study, calf spleen-derived PNP was used to treat 12.5 ºP wort, resulting in the degradation of 60% of purine nucleosides in the wort [8]. Recombinant PNP from various sources has been successfully expressed in *E. coli* and shown to demonstrate high enzyme activity. For instance, bovine PNP expressed in *E. coli* had a specific activity of 30 U/mg [10], while human PNP expressed in *E. coli* achieved a 707-fold increase in enzyme activity compared to the control [11]. Nevertheless, there has been limited research on PNP from food-grade microorganisms.

*Kluyveromyces lactis* is a microorganism deemed Generally Recognized as Safe (GRAS) by the U.S. Food and Drug Administration (FDA) [12]. Thus, its enzyme system has potential applications in the food industry. It was successfully expressed as *K. lactis* KlacPNP in *E. coli* and mutated to KlacPNP (N256D) [13]. The enzyme shows optimal activity at 25 °C and only maintains about 30% of its activity at the higher temperature limit of 45 °C. However, the temperature range during wort saccharification is between 45 and 78 °C, so its use in the beer industry is still limited.

*Pichia pastoris*, a widely used protein expression system second only to *E. coli*, is commonly employed for laboratory-based protein production, characterization, and structural analysis. It has also been recognized as a GRAS microorganism by the U.S. FDA, paving the way for its application in the food and pharmaceutical sectors. Currently, there are no reports on the heterologous expression of PNP in eukaryotic systems. Large edible fungi are known to be rich in purine analogs. Among them, *Agaricus bisporus* can utilize urate, allantoin, allantoic acid, and urea as nitrogen sources for its growth [14]. This implies that *A. bisporus* not only meets food safety requirements but also contains a well-developed system of purine-degrading enzymes. Therefore, the purine-metabolizing enzyme system of *A. bisporus* demonstrates potential applications in the beer industry.

Here, we report the first identification and characterization of a PNP from *A. bisporus* (*Ab*PNP) and its application in low-purine beer production. Key objectives include (1) heterologous expression of *Ab*PNP in P. pastoris; (2) biochemical characterization of *Ab*PNP’s pH/thermal stability and kinetic parameters; (3) evaluation of purine reduction efficacy in wort saccharification; and (4) analysis of fermentation performance and flavor impacts. This strategy will provide various low-purine-content foods and beverages for patients without the risk of gout and hyperuricemia.

## 2. Materials and Methods

### 2.1. Plasmids and Strains

After codon optimization, the *Ab*PNP gene was synthesized by Genewiz (Suzhou, China) based on the *A. bisporus* var. burnettii JB137-S8 PNP gene (NCBI GenBank accession no. XM_007331084). Then, the acquired sequences were submitted to GenBank (accession ON012779). The synthetic and wild-type genes were inserted between pPIC9K plasmid’s EcoRI and NotI sites to acquire recombinant pPIC9K-*Ab*PNP containing a 6 × His tag coding sequence at the N-terminus. DNA sequencing was carried out to confirm the recombinant plasmid. The brewer’s yeast strain SC4 (Ale yeast) was acquired from the National Engineering Research Center of Cereal Fermentation and Food Biomanufacturing, Jiangnan University (Wuxi, China).

### 2.2. Structural Bioinformatics Analysis

The *A. bisporus* PNP (*Ab*PNP) model (NCBI accession nos. XP007331146) was created with the help of the Swiss Model (https://swissmodel.expasy.org/ accessed on 10 February 2022) using the PNP from *Bos taurus* (PDB ID: 3FUC) as a template. Molecular mass was estimated using ExPASy (https://www.expasy.org/ accessed on 10 February 2022). For sequence alignment, the ESPript server was employed (https://espript.ibcp.fr/ESPript/ESPript/ accessed on 10 February 2022). The phylogenetic tree was constructed using the MEGA 11.0 program with the neighbor-joining algorithm and 1000 bootstrap replicates, based on sequences downloaded from the NCBI database, including the yeast species *K. lactis* (XP452943) and *Saccharomyces cerevisiae* (NP013310), the mammalian species *B. taurus* (NP001007819) and *Homo sapiens* (NP000261), the filamentous fungi species *Lyophyllum atratum* (KAF8056904) and *Leucoagaricus* sp. (KXN89711), and the bacterial species *Pseudoalteromonas* sp. (ABN13115) and *E. coli* (WP073446691).

### 2.3. Expression and Purification of PNPs in P. pastoris

The standard protocol of Invitrogen, Waltham, MA, USA (Original Pichia Expression Kit) was followed to prepare GS115 yeast electrocompetent cells. pPIC9K-*Ab*PNP was linearized utilizing restriction endonuclease *Sal* I, and the product was acquired via a gel extraction kit. Then, the linearized plasmid (10 μL) was transformed into electrocompetent cells (100 μL). Subsequently, the transformed cells were grown on the MD plates [glucose (2% *w*/*v*), yeast nitrogenous base lacking amino acids (1.34% *w*/*v*), biotin (0.00004% *w*/*v*), and agar (1.5% *w*/*v*)] at 30 °C for 3–5 days until individual colonies were observed. The colonies were then marked, isolated, and cultured in MD and MM [biotin (0.00004% *w*/*v*), yeast nitrogenous base lacking amino acids (1.34% *w*/*v*), agar–agar (1.5% *w*/*v*), and methanol (0.5% *v*/*v*)] plates at 30 °C for 2–3 days. The *P. pastoris* transformants only propagated on the MD plates. The genomic DNA of these transformants (randomly selected) was extracted per the standard method (Yeast DNA Extraction Kit, Invitrogen) and screened using 5′AOX1 and 3′AOX1 primers (5′AOX: GACTGGTTCCAATTGACAAGC; 3′AOX: GGCAAATGGCATTCTGACATCCT) [15]. The genomic DNA was then employed as a template for PCR amplification. The PCR conditions were as follows: initial denaturation cycle at 95 °C for 3 min, and then 30 cycles of 30 s at 95 °C for denaturation, 30 s at 55 °C for annealing, and 90 s at 72 °C for elongation, and a last step of elongation at 72 °C for 10 min. Lastly, the amplified product was subjected to agarose electrophoresis, and the correct transformants were selected for subsequent studies.

From the MD plates, a *P. pastoris* colony GS115 pPIC9K-*Ab*PNP/pPIC9K-*Ab*PNP-wt was inoculated into 5 mL of BMGY [peptone (2% *w*/*v*), yeast extract (1% *w*/*v*), 0.1 M potassium phosphate (10% *v*/*v*), pH 6.0, glycerin (0.1% *v*/*v*), yeast nitrogenous base lacking amino acids (1.34% *w*/*v*), and biotin (0.00004% *w*/*v*)] for 48 h at 30 °C. The selected colony was centrifuged for 5 min at 5000 rpm to collect cellular pellet, which was resuspended in 1 mL of BMMY [peptone (2% *w*/*v*), yeast extract (1% *w*/*v*), 0.1 M potassium phosphate (10% *v*/*v*), pH 6.0, methanol (0.5% *v*/*v*), yeast nitrogenous base lacking amino acids (1.34% *w*/*v*), and biotin (0.00004% *w*/*v*)] medium and cultured for 4 days at 30 °C. Then, protein expression was induced, which was maintained by adding methanol (1% *v*/*v*) once daily. Subsequently, the samples were collected and centrifuged to harvest pellets, which were analyzed by 12% SDS-PAGE.

The gradient elution method was followed for enzyme purification via Ni-NTA affinity column chromatography [16]. Furthermore, the HisTrap HP column (1 mL, GE Healthcare, Chicago, IL, USA)-equipped AKTA purifier was also utilized. The protein was eluted over a linear imidazole gradient from 20 to 500 mM in the buffer. The purified enzyme was acquired in 200 mM imidazole and then desalted using a PD-10 desalting column.

### 2.4. Enzyme Activity Assay

*Ab*PNP’s activity was investigated according to the phosphorolysis reaction from inosine to hypoxanthine [17]. The enzyme activity unit is defined as the amount of enzyme required to produce 1 μmol of hypoxanthine per minute under the reaction conditions of 37 °C and pH 7.0. To acquire hypoxanthine, the assay mixture [potassium phosphate buffer (50 mM; pH 7.0), inosine (10 mM), and *Ab*PNP (500 μL)] was incubated at 37 °C for 10 min and then measured by high-performance liquid chromatography (HPLC).

### 2.5. Biochemical Characterization of AbPNP

The impact of pH (from 4.0 to 11.0) on the function of *Ab*PNP was elucidated at 37 °C for 10 min using inosine (10 mM) as the substrate. For the pH stability analysis, the enzyme was treated with 3 buffer solutions, including K_2_HPO_4_-KH_2_PO_4_ (pH 6.0–8.0; 50 mM), sodium citrate–citric acid (pH 4.0–6.0; 50 mM), and glycine-NaOH (pH 8.0–11.0; 50 mM) for 2 h at 4 °C. The optimum temperature was evaluated by starting the reaction at various temperatures from 20 to 90 °C. Furthermore, for thermostability analysis, *Ab*PNP was incubated at 30–80 °C temperatures for up to 180 min before the residual activity detection. The initial activity was considered 100%. Moreover, the impact of different chemical reagent cations and metal ions on the *Ab*PNP function was elucidated per the aforementioned standard assay parameters. The reaction mixture was mixed with effectors (Mg^2+^, K^+^, Fe^2+^, Fe^3+^, Ni^2+^, Al^3+^, Co^2+^, Li^+^, Ca^2+^, Zn^2+^, Cu^2+^, Urea, Mn^2+^, and EDTA) at a final concentration of 5 mM. A blank lacking chemical reagents and extra metal ions was also assayed. Kinetic parameters (Km and Vmax and) were evaluated at pH 7.0 and 60 °C optimal temperature employing 0–16 mM of inosine as substrate. All the experiments were carried out in triplicates. The classical Michaelis–Menten equation was utilized for assessing the turnover number (kcat) and apparent Km.

### 2.6. Wort Preparation

The mashing protocol was as follows: gelatinization (45 °C for 30 min), 63 °C for 40 min, saccharification (72 °C for 20 min), and total starch gelatinization (78 °C for 10 min). The temperature ramp-up heating rate was 1 °C/min. Furthermore, the Canadian barley malt (50 g; COFCO Corporation) was milled and mashed in a mashing bath (BGT-8A, Bioer) at a water-to-malt ratio of 4:1. The *Ab*PNP enzymes (1.0–5.0 U/mL) were added at the 45 °C holding stage. After the mashing process, the wort was filtered and boiled with hops (0.3%; α-acid content = 6%) for 60 min. The acquired mixture was filtered again and adjusted to 12 °Plato. Control experiments were performed without *Ab*PNP, and all experiments were repeated three times.

### 2.7. Wort Fermentation

The brewer’s yeast strain SC4 was pre-cultured for 2 days in the wort (100 mL) at 28 °C and then harvested after 12 h of natural sedimentation at 4 °C. For primary fermentation, the wort (300 mL) was inoculated with yeast (10^7^ cells/mL) at 20 °C for 5 days. Using the airlocks, the flasks were closed. Three replications were performed using the brewer’s yeast strain SC4.

### 2.8. Determination of Total Purine Content

The samples were degassed and filtered through a 0.22 μm aqueous membrane filter prior to analysis using HPLC [5]. The HPLC system (Agilent 1260) was equipped with an Agilent ZORBAX Eclipse XDB-C18 column (250 × 4.6 mm, 5 μm) and operated under the following conditions: mobile phase A (water-glacial acetic acid-tetrabutylammonium hydroxide, 991.5:7:1.5, *v*/*v*/*v*) and mobile phase B (methanol) mixed at a ratio of 90:10, flow rate of 1 mL/min, injection volume of 20 μL, detection wavelength of 254 nm, and column temperature of 30 °C. Seven purine components—adenine (A), guanine (G), hypoxanthine (H), xanthine (X), inosine (Ino), guanosine (Gua), and adenosine (Ade)—were quantified based on their retention times and standard curves, with TP calculated as the sum of these components.

For standard curve preparation, stock solutions of the seven purines (100 mg/L) were serially diluted to concentrations of 0.1–100.0 mg/L. Each concentration was analyzed in triplicate by HPLC, and calibration curves were generated by plotting peak areas against corresponding concentrations.

### 2.9. Analytical Methods

The real degree of fermentation (RDF), original extract, and alcohol concentration in terminal fermentation samples were quantified using a density meter (DMA 5000 M, Anton Paar GmbH, Graz, Austria) with integrated temperature control. Concurrently, volatile compounds (acetaldehyde, higher alcohols, and esters) were determined through static headspace gas chromatography (HS-GC) employing a Clarus 500 GC system (PerkinElmer, Waltham, MA, USA) coupled with an HS40 automated sampler. The analytical separation was achieved using a fused-silica DB-5 capillary column (60 m × 0.53 mm ID, 1.5 μm film thickness; Agilent, Santa Clara, CA, USA) maintained under optimized temperature-programmed conditions, with compound identification verified through external calibration curves established with certified reference standards [18].

### 2.10. Statistical Analysis

All tests were conducted in triplicate, and the results are presented as the mean ± standard error. To determine if two sets of data were significantly different from each other, an unpaired two-sample Student’s t-test was performed using SPSS software (version 23.0, SPSS Inc., Chicago, IL, USA). A *p*-value of less than 0.05 was regarded as statistically significant. Additionally, all calculations were executed with GraphPad Prism Software 9.0 (GraphPad Software, Inc., San Diego, CA, USA).

## 3. Results and Discussion

### 3.1. The Analysis of Amino Acid Structure and Sequence

**Figure 1:**
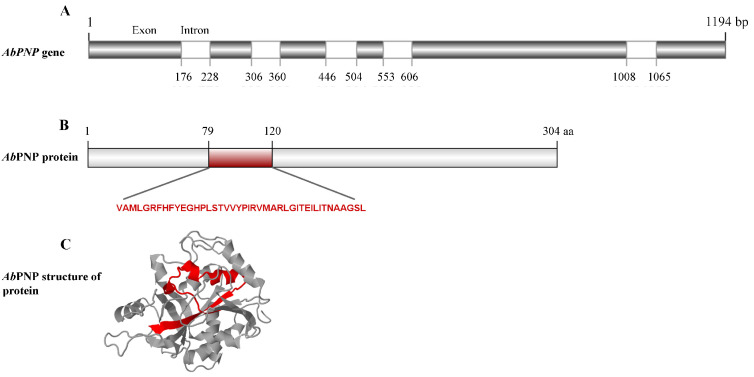
The sequence characteristics of *Ab*PNP. (**A**). The gene structure of *AbPNP*; (**B**). The protein structure of *Ab*PNP; (**C**). *Ab*PNP protein three-dimensional structure.

The *Ab*PNP putative gene (Gene ID:18825793) has a sequence length of 1194 bp and contains five introns (Figure 1A) located at 176–228 bp, 306–360 bp, 446–504 bp, 553–606 bp, and 1008–1065 bp, with a coding sequence length of 915 bp; it encodes for *Ab*PNP containing 304 amino acid residues, and its motif is between amino acid residues 79 and 120 (Figure 1B).The *Ab*PNP amino acid sequence was submitted to Expasy and SignalP websites for analysis, and the results showed that *Ab*PNP was expressed intracellularly without a signal peptide, with a molecular weight of approximately 32.52 kDa and a theoretical isoelectric point pI of 5.85.

**Figure 2:**
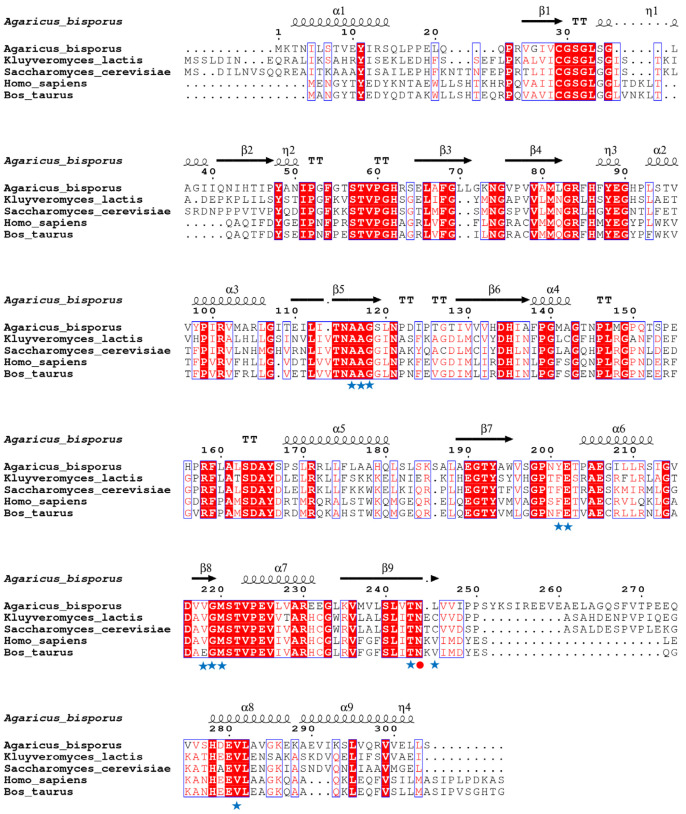
Amino acid sequence alignment of PNP using ESPript server. Highly conservative regions are highlighted with red boxes and conservative substitutions are shown with boxes. Active site residues involved in hypoxanthine action are shown with asterisks, and catalytically active residues known to play an important role in substrate specificity are shown with red-filled circles.

**Figure 3:**
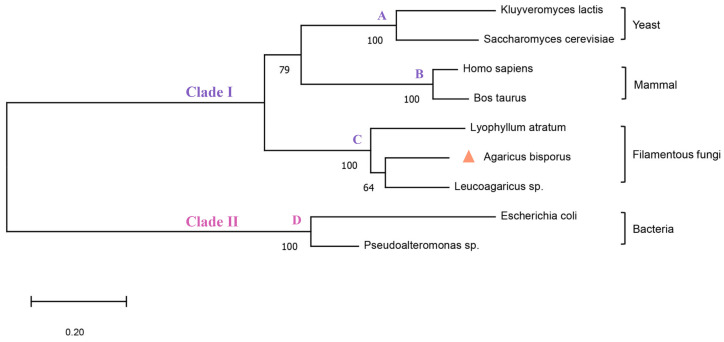
Phylogenetic analysis of PNPs. Group A belongs to yeasts, Group B to mammals, Group C to filamentous fungi and Group D to bacteria. The triangular symbols represent the dictyostelium mushrooms used in this study.

The calf PNP’s experimentally dissolved X-ray structure (PDB ID: 3FUC) was employed as the template as it shares 43.5% sequence identity with *Ab*PNP and significantly matches the region near the active site (Figure 2). The *Ab*PNP’s phosphate-binding site primarily contained G30, S31, H62, R84, H86, N115, A116, and S221, and the purine nucleoside binding sites were S31, H86, Y88, A116, A117, G118, Y201, E202, V218, G219, M220, T243, N244, H278, and V281. The eight PNP protein sequences were analyzed for homology using the neighbor-joining method for homology analysis and construction of a phylogenetic tree (Figure 3). Eukaryotic and prokaryotic PNPs were clustered into two clades, I and II, respectively. Clade I included yeast, mammals, and filamentous fungi, in which *Ab*PNPs were clustered into a group of filamentous fungi; Clade II included bacteria. The above sequence analyses and structure predictions confirmed the putative gene PNP from *A. bisporus* as a purine nucleoside phosphorylase gene.

### 3.2. Expression of Recombinant AbPNP in P. pastoris

**Figure 4:**
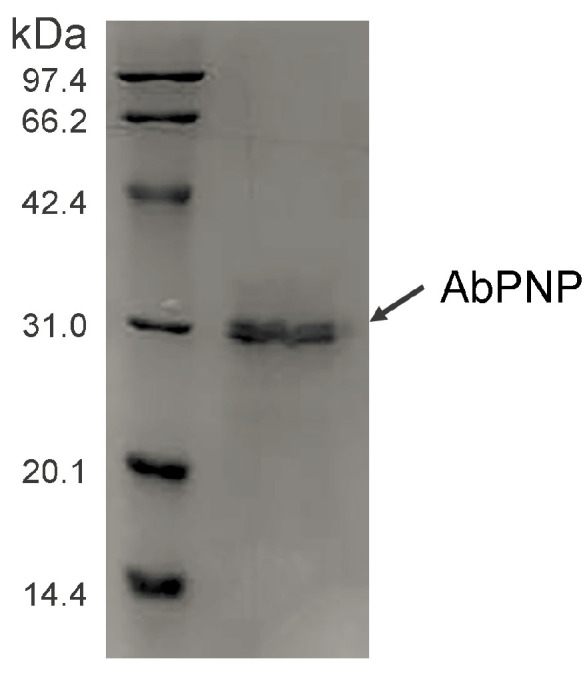
SDS-PAGE analysis of *Ab*PNP protein.

The codon-optimized *Ab*PNP gene was successfully expressed in *P. pastoris*. SDS-PAGE indicated a specific band of about 32.5 kDa in the purified sample (Figure 4), which corresponds well to *Ab*PNP’s molecular weight assessed via gene sequence. Furthermore, *Ab*PNP’s molecular mass was consistent with that reported previously, such as *Bos taurus* PNP (31.6 kDa) [19], *K. lactis* PNP (33.4 kDa) [13], *Saccharomyces cerevisiae* PNP (33.7 kDa), *Homo sapiens* PNP (32.1 kDa), *Lyophyllum atratum* PNP (33.6 kDa), and *Leucoagaricus* sp. PNP (32.6 kDa). These data indicated that the *Ab*PNP protein was successfully expressed in *P. pastoris* and secreted into the culture.

### 3.3. Enzymatic Properties of AbPNP

**Figure 5:**
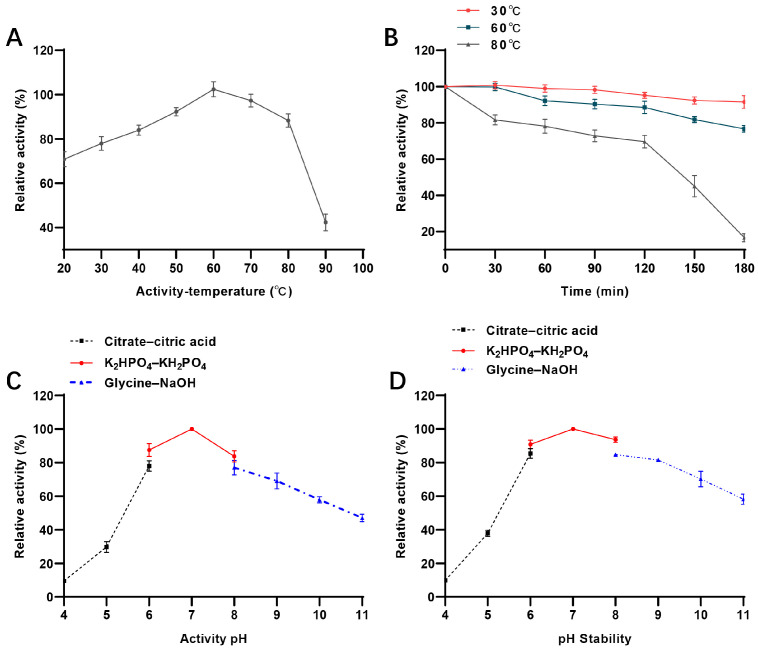
Enzymatic properties of recombinant AbPNP. (**A**) optimum temperature; (**B**) temperature stability; (**C**) optimum pH; (**D**) pH stability.

Temperature also crucially influences enzyme applications. Here, it was observed that *Ab*PNP was active over a wide range of temperatures (20–90 °C). The best activity was observed at 60 °C (Figure 5A), similar to that of *E. coli* [20] and higher than that of *K. lac* (25 °C) [13], *H. sapiens* (25 °C) [21], *B. taurus* (25 °C) [22], and *S. cerevisiae* (30 °C) [23]. Moreover, *Ab*PNP was more stable at temperatures ≤ 60 °C (Figure 5B), retaining at least 85% of its activity after 3 h of incubation. Further, it indicated a half-life of 150 min at 80 °C, with decreasing stability at 90 °C where it retained only 20% of its activity after 30 min of incubation. *Ab*PNP’s thermal stability was superior to that of the most reported PNP, i.e., 50 °C. *K. lac* PNP from a food-grade source is moderately thermostable with only 30% activity after 2 h of incubation at 45 °C. During beer brewing, since the saccharification stage temperature rises between 45–72 °C, high thermal stability is required, and *Ab*PNP meets this criterion.

The impact of pH, temperature, and metal ions on the enzymatic properties of *Ab*PNP was also characterized. The data revealed that *Ab*PNP had high activity between pH 5.0 and 11.0, with an optimal reaction pH of 7.0 (Figure 5C), comparable to PNPs of *H. sapiens* (7.0) [21], *B. taurus* (7.0) [22], and *S. cerevisiae* (7.5) [23]. Furthermore, *Ab*PNP was stable in the 6.0–9.0 pH range, retaining >80% of its initial activity after incubation for 2 h at 4 °C (Figure 5D). Therefore, it was inferred that *Ab*PNP had significant stability and could be applied in food industries, especially the beer industry.

**Figure 6:**
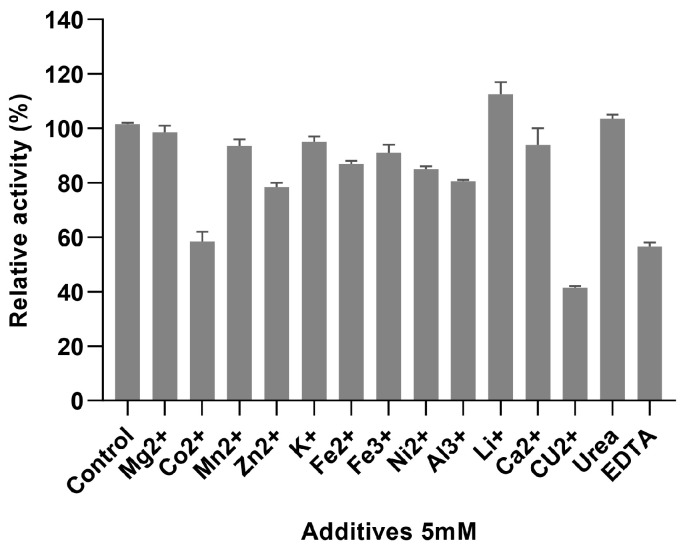
The effect of additives on the activity of *Ab*PNP.

These thermal properties of *Ab*PNP also make it suitable for potential applications in the beverage industry. At 5 mM metal ion and chemical reagent concentration (Figure 6), Co^2+^, Cu^2+^, and EDTA had a significant inhibitory effect on *Ab*PNP, with residual activities of 58.50 ± 4.95%, 41.50 ± 0.71%, and 56.50 ± 1.21%, respectively. Furthermore, Zn^2+^ and Al^3+^ indicated some inhibitory effect, with 78.50 ± 2.12% and 80.50 ± 0.71% residual activities, respectively. Li^+^ showed a promoting effect on the *Ab*PNP activity (112.50 ± 6.36%), whereas other metal ions and urea indicated minimal effect. Li et al. [24] showed that in the wort, metal ions were mainly acquired from water and malt, including Mg^2+^ (3.5–4.0 mmol/L), Na^+^ (0.9–2.0 mmol/L), Fe^3+^ (0.003–0.005 mmol/L), and Ca^2+^ (0.75–1.0 mmol/L). The present study found no effect of these metal ions on *Ab*PNP. Zn^2+^ is a crucial factor for yeast growth; it is usually added before the inoculation of brewer’s yeast and is not affected by *Ab*PNP when applied to wort saccharification. These results indicate that the *Ab*PNP is highly resistant to most metal ions.

### 3.4. Substrate Specificity and Enzyme Kinetics

Due to the high purine nucleoside content in the wort, the purine content of beer can be effectively reduced by degrading purine nucleosides. The specificity of *Ab*PNP substrates was determined using adenine, guanine, and hypoxanthine nucleosides. Recombinant *Ab*PNP indicated different degradation capabilities for hypoxanthine and guanine nucleosides. The highest enzyme activity was measured when hypoxanthine nucleoside was used as a substrate (100%), followed by guanine nucleoside (55.30 ± 3.25%), whereas no activity was observed with adenine nucleoside. Similar to mammals, PNP showed no activity for 6-aminopurine nucleosides (Ado) and specificity for 6-oxopurine nucleosides (Guo and Ino).

The Michaelis–Menten kinetics of *Ab*PNP with inosine as substrate were normal. At pH 7.0 and 60 °C, the respective Km and kcat values of *Ab*PNP were 7.1 µmol and 14.38 s^−1^, with a catalytic efficiency of 2.02 × 10^6^ s^−1^·M^−1^. AbPNP’s Km values were lower than those of *K. lac* (21.0 µmol/L) [13], *B. taurus* (13.4 µmol/L) [10], and *H. sapiens* (30 µmol/L) [25]. Furthermore, *Ab*PNP’s Km value was higher than that of PNP of *Pseudoalteromonas* sp. (0.39 µmol/L) [26]; however, its kcat/Km was 2.15 × 10^5^ s^−1^·M^−1^, which was lower than that of *Ab*PNP (2.02 × 10^6^ s^−1^·M^−1^) in this study. The smaller the Km value, the stronger its substrate affinity and the specific activity, indicating that the enzyme will more effectively degrade the substrate at low concentrations, and the low enzyme usage has a very important potential for application in the food industry.

### 3.5. Nucleoside Degradation Analysis in the Mashing Process

**Figure 7:**
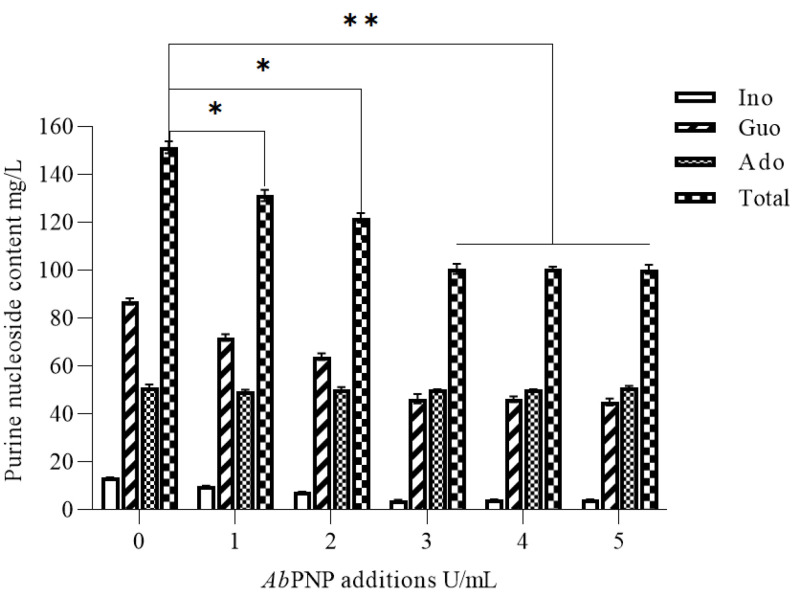
The effect of *Ab*PNP on free purine nucleoside content in the wort of barley malts. * indicates a significant difference, *p* < 0.05; ** indicates a highly significant difference, *p* < 0.01.

The *Ab*PNP’s temperature and pH stability met the requirements of the saccharification phase and retained > 80% of its activity at 60 °C after 180 min. The literature has revealed that the timing of PNP addition did not substantially impact purine nucleoside degradation in the wort [27]. Therefore, in this study, *Ab*PNP (1.0–5.0 U/mL) was added at the saccharification (45 °C) onset to maximize the purine nucleoside degradation. The results showed that the total purine nucleosides in the wort obtained after *Ab*PNP addition were highly significantly reduced by 33.58% from 151.53 to 100.65 mg/L (*p* < 0.01) (Figure 7). Since PNP is a reversible enzyme when >3 U/mL *Ab*PNP was added, the degradation reaction reached an equilibrium state, and the total purine nucleosides remained unchanged.

### 3.6. The Effect of AbPNP on Physicochemical Parameters and Purines of Beer

The total free purine base content in the beer brewed from the enzyme-treated wort (33.54 mg/L) was higher than that of the control (26.87 mg/L), with a significant increase (*p* < 0.05) in guanine (10.34 mg/L), hypoxanthine (6.71 mg/L), and xanthine (9.90 mg/L). It might be due to the limited ability of brewer’s yeast to utilize free purine bases. The total nucleoside content decreased from 151.53 to 100.65 mg/L, and the content of hypoxanthine nucleoside and guanine nucleoside both decreased significantly (*p* < 0.05). In conclusion, the addition of *Ab*PNP during wort saccharification significantly reduced the purine nucleoside content in beer.

Table 1 indicates the physicochemical indexes of the beer brewed from the wort and prepared by the addition of *Ab*PNP during saccharification. The data show that the alcohol content of the beer increased by 3.57% from 4.48% to 4.64% vol (*p* < 0.05). Furthermore, the RDF increased from 71.79% to 73.68%, showing an increase of 2.63%. Moreover, the total purine nucleoside levels were reduced by 33.58%, which was primarily because of more free purine bases available to the yeast, which, in turn, elevated proliferative capacity and fermentation performance, consistent with increased alcoholic strength and fermentation degree in beer samples.

### 3.7. The Effect of AbPNP on Beer Aroma Compounds

In order to assess the main flavor characteristics of the beer, the content of the main higher alcohols and volatile esters in the beer was determined in this study (Table 2). Advanced alcohols and esters are the main volatile components of beer and are key determinants of the final quality of the beer, with the appropriate ratio being between 4.0 and 5.0.

The addition of *Ab*PNP resulted in elevated concentrations of total higher alcohols (136.12 mg/L) and total esters (30.92 mg/L) compared to the control group (119.67 mg/L higher alcohols and 29.24 mg/L total esters). Notably, isoamyl alcohol, phenethyl alcohol, and isoamyl acetate exhibited statistically significant increases (*p* < 0.05). Propanol, primarily derived from pyruvate metabolism or glycolytic intermediates, showed a marginal elevation potentially attributed to enhanced yeast metabolic activity and subsequent pyruvate flux following PNP enzyme supplementation. However, its low proportion in beer resulted in non-significant variation.

Isoamyl alcohol, the predominant higher alcohol in beer [28], originates predominantly from the Ehrlich pathway of leucine catabolism. Enhanced yeast metabolism and improved fermentation efficiency significantly promoted the generation of isoamyl alcohol and phenethyl alcohol. Ethyl acetate synthesis via ethanol and acetyl-CoA was accelerated by increased acetyl-CoA production and elevated ethanol concentrations [29], contributing to intensified fruity aromas. The substantial increase in isoamyl acetate likely resulted from abundant isoamyl alcohol availability and sufficient acetyl-CoA supply.

Ethyl hexanoate formation through hexanoic acid and ethanol esterification was enhanced by improved medium-chain fatty acid metabolism and ethanol-driven esterification, imparting sweet or apple-like notes [30]. Ethyl caprylate content showed moderate elevation, potentially associated with accelerated long-chain fatty acid metabolism and ethanol-mediated esterification. Importantly, *Ab*PNP supplementation did not significantly alter the proportion between higher alcohols and volatile esters, with the alcohol-to-ester ratio increasing from 4.09 to 4.40, remaining within acceptable parameters.

## 4. Conclusions

This study successfully cloned a novel purine nucleoside phosphorylase gene, *Ab*PNP, from *A. bisporus* and achieved efficient heterologous expression in the *P. pastoris* GS115 system. The recombinant *Ab*PNP exhibited optimal catalytic activity at 60 °C and pH 7.0 Its thermal stability (maintaining >85% activity after 3 h at 60 °C) and broad pH tolerance (maintaining >80% activity at pH 6.0–9.0) are significantly superior to traditional PNP derived from bovine spleen and *K. lac*. Enzyme kinetic analysis revealed that *Ab*PNP exhibits high affinity (Km = 7.10 μM) and catalytic efficiency (kcat/Km = 2.02 × 10^6^ s^−1^·M^−1^) for the substrate inosine and demonstrates good resistance to metal ions other than Co^2+^ and Cu^2+^. In the beer brewing system, *Ab*PNP effectively degrades purine nucleosides in the wort by converting nucleosides into free bases that can be utilized by yeast, thereby reducing the purine content in beer. This food-grade enzyme derived from the genes of an edible fungus offers excellent process compatibility and safety, providing an innovative solution for the development of low-purine beers aimed at preventing gout and hyperuricemia. Follow-up research will focus on optimizing the industrial scale of production and expanding its application in other fermented foods.

## Figures and Tables

**Table 1:** Basic physicochemical characteristics and purines of beers.

Parameters	CK	*Ab*PNP Addition
Alcohol %/vol	4.48 ± 0.05	4.64 ± 0.03 *
RDF/%	71.79 ± 0.42	73.68 ± 0.51
Original extract/% *w*/*w*	12.20 ± 0.15	12.21 ± 0.09
Adenine/mg/L	7.23 ± 0.13	6.59 ± 0.24
Guanine/mg/L	5.04 ± 0.06	10.34 ± 0.19 *
Hypoxanthine/mg/L	5.81 ± 0.12	6.71 ± 0.08 *
Xanthine/mg/L	8.79 ± 0.57	9.90 ± 0.14 *
Total purine bases/mg/L	26.87	33.54
Inosine/mg/L	13.35 ± 0.11	4.12 ± 0.04 *
Guanosine/mg/L	87.88 ± 3.76	46.38 ± 2.14 *
Adenosine/mg/L	50.30 ± 2.16	50.15 ± 1.47
Total purine nucleoside/mg/L	151.53	100.65

Note: Values are the mean ± standard deviations in three independent fermentations, and asterisks indicate significant differences in the addition or non-addition of *Ab*PNP during wort saccharification, as determined by a two-tailed Student’s *t*-test (* *p* < 0.05).

**Table 2:** The concentration of aroma compounds of beers (mg/L).

Aroma Compounds	CK	*Ab*PNP Addition
Propanol	13.08 ± 0.49	13.20 ± 0.64
Isobutanol	26.38 ± 0.77	26.51 ± 2.11
Isoamyl alcohol	51.04 ± 3.41	60.73 ± 3.32 *
Phenethyl alcohol	29.17 ± 2.86	35.68 ± 1.78 *
Total higher alcohols	119.67	136.12
Ethyl acetate	25.86 ± 2.39	27.01 ± 1.54
Isoamyl acetate	2.97 ± 0.11	3.47 ± 0.09 *
Ethyl hexanoate	0.22 ± 0.01	0.23 ± 0.01
Ethyl caprylate	0.19 ± 0.01	0.21 ± 0.01
Total esters	29.24	30.92
Ratio of total higher alcohols to esters	4.09	4.40

Note: Values are the mean ± standard deviations in three independent fermentations, and asterisks indicate significant differences in the addition or non-addition of *Ab*PNP during wort saccharification, as determined by a two-tailed Student’s *t*-test (* *p* < 0.05).

## Data Availability

The original contributions presented in this study are included in the article. Further inquiries can be directed to the corresponding author.
